# Total Lipid Extracts of Honeybee Drone Larvae Are Modulated by Extraction Temperature and Display Consistent Anti-Inflammatory Potential

**DOI:** 10.3390/foods12224058

**Published:** 2023-11-08

**Authors:** Yiming Luo, Yuyang Guo, Wen Zhao, Shaden A. M. Khalifa, Hesham R. El-Seedi, Xiaoling Su, Liming Wu

**Affiliations:** 1 State Key Laboratory of Resource Insects, Institute of Apicultural Research, Chinese Academy of Agricultural Sciences, Beijing 100093, China; 82101215462@caas.cn (Y.L.); guoyuyang1026@163.com (Y.G.); zhaowen1309@126.com (W.Z.); 2 School of Life Science, Liaocheng University, Liaocheng 252059, China; 3 Psychiatry and Psychology Department, Capio Saint Göran’s Hospital, Sankt Göransplan 1, 11219 Stockholm, Sweden; shaden.khalifa@regionstockholm.se; 4 Department of Chemistry, Faculty of Science, Islamic University of Madinah, Madinah 42351, Saudi Arabia; 5 International Research Center for Food Nutrition and Safety, Jiangsu University, Zhenjiang 212013, China; 6 Jinhua Academy of Agricultural Sciences, Jinhua 321000, China

**Keywords:** honey bees, drone larvae, anti-inflammatory, UPLC-Q-exactive-orbitrap–MS, GC–MS QP2010plus, lipidomics

## Abstract

Honeybee drone larvae are male bees that develop from unfertilized eggs and play a role in colony reproduction. The nutritional value of honeybee drone larvae is due to their high protein, lipid, and other nutrient contents, making them a profitable food source for humans in some cultures. Drone larvae lipids (DLLs) contribute to drone development; however, few studies have explored their substantial compositions and bioactive functions. In this study, we carried out DLL lipidomics analysis using UPLC-Q-Exactive-Orbitrap–MS prior to in vitro anti-inflammatory activity analysis. The results highlighted the importance of the extraction temperature on the DLL composition. A total of 21 lipids were found in the DLL extract, mostly categorized into five groups: nine phospholipids, three sphingolipids, two neutral lipids, one plant glycoglycerolipid, four lipid acyl, and others. Drying extraction at −20 °C produced more sphingolipids, phospholipids, and unsaturated fatty acids. Of 37 fatty acids, 18 were displayed at −20 °C degrees, as shown by GC–MS quantitative analysis. Myristic (246.99 ± 13.19 μg/g), palmitic (1707.87 ± 60.53 μg/g), stearic (852.32 ± 24.17 μg/g), and oleic (2463.03 ± 149.61 μg/g) acids were the predominant fatty acids. Furthermore, we examined the significant in vitro anti-inflammatory effects of DLL (−20 °C) using lipopolysaccharide (LPS)-challenged RAW264.7 cells. Nitric oxide (NO) and reactive oxygen (ROS) production and mRNA expression of IL-6, IL-10, COX-2, and iNOS were significantly decreased, demonstrating the anti-inflammatory function of DLL. Overall, this study provided insight into the lipid composition of DLL, revealed the influence of temperature, and explored the functionality of DLL (−20 °C), allowing for further application of DLLs as functional foods.

## 1. Introduction

Investment in the field of functional foods is a crucial driver for the food business that changes the future of food production and consumption [1,2,3]. Functional foods are defined as those that function beyond their fundamental nutritional requirements to improve human health and quality of life [4,5,6,7]. The development of value-added and health-oriented foods is therefore becoming increasingly popular.

Drones, as male honeybees, are one of the polymorphic forms of bees produced from unfertilized eggs, with the ability to reproduce and play a crucial role in ensuring the survival and growth of colonies. Their primary function is to mate with a receptive queen bee, thereby ensuring the production of future generations of honey bees and facilitating the creation of new colonies [8,9]. Honeybee larvae can be utilized as food for humans as well as livestock in a variety of regions, while beekeepers can earn additional income by collecting and processing them. In general, the amino acid composition and total protein concentration increased as the drone developmental stage progressed. Conversely, the amount of fatty acids decreased as the drone matured. Late developmental stages exhibited higher concentrations of most minerals. Therefore, drones are a suitable nutritional resource due to their protein content (31.4–43.4%), fat content (9.5–11.5%), and abundance of minerals such as iron and zinc [10]. Although pupae contain more nutrients, particularly protein, than prepupae (larvae aged from 10 to 14 days), given the latter’s larger biomass, we recommend the use of prepupae (larvae aged 10–14 days) as a commercial product. However, unlike queen larvae, worker larvae, and drone pupae, which have both established substantial industrial chains for the manufacturing of health-related products, drone larvae have not yet begun to see widespread commercial development [11]. Several scientific studies support the remarkable value of drone larvae in treating a wide range of illnesses, including malnutrition, ovarian dysfunction, and infertility [12,13,14]. Drone larvae lipids (DLLs) are thought to play an essential role. However, few studies on the components of drone larvae, particularly on lipids, are still in their infancy. Hence, more research is warranted to fully understand the chemical and biological basis.

Lipids play beneficial roles in antibacterial, antioxidant, anti-inflammatory, antihypertensive, antiaging, anticancer, antidiabetic, wound healing, hypolipidemic, and immunological modulation [15]. The contributions of dietary lipids to a healthy lifestyle have been widely explored in recent years [16]. Many epidemiological and clinical trials have shown a positive link between consuming omega-3 polyunsaturated fatty acids and the release of inflammation [17]. Traditionally, lipid composition analysis is performed by chromatographic techniques combined with appropriate detectors following esterification and derivatization [18,19]. The improvement of lipid identification technologies coincides with the advancement of lipidomics. Since its inception in 2003, lipidomics has improved significantly, partly as a result of the advancement of mass spectrometry (MS) [20,21]. HPLC-MS, or MS/MS, has been widely used in lipidomics based on its good reproducibility, high resolution, sufficient separation of isomers, comprehensive analysis of complex samples, meeting the different needs of various lipids/species, and high sensitivity [22,23,24].

Thus, the aim of this paper was to further explore the drone larvae and focus on their lipid composition and anti-inflammatory activity by applying up-to-date analytical strategies. This could potentially boost the usage of drones as an additional product for beehives, ultimately benefiting the economy of small to medium-sized beekeepers.

## 2. Materials and Methods

### 2.1. Chemicals and Reagents

Methanol, HPLC-grade water, acetonitrile, and isopropanol were purchased from Fisher Scientific Inc. (Pittsburgh, PA, USA). Ammonium formate, ammonium acetate, and ammonium hydroxide solutions were purchased from Sigma-Aldrich (Saint Louis, MO, USA). Formic acid was purchased from Dikma Technologies (Foothill Ranch, CA, USA), and methylene chloride was purchased from Amresco (Solon, OH, USA). The standards for phosphatidylcholine (PC), phosphatidylethanolamine PE, phosphatidylglycerol (PG), phosphatidylserine (PS), and lysophosphatidylcholine (LPC) were obtained from Avanti Inc. (Shelby, AL, USA), and ceramide (Cer), diacylglycerol (DG), triacylglycerol TG, and fatty acid (FA) were obtained from Sigma-Aldrich Inc. (Saint Louis, MO, USA). All solvents for lipid extraction or used as mobile phases were of chromatographic grade. Ultrapure water was obtained from a Millipore Milli-Q water purification system (Millipore, Bedford, MA, USA). LPS (*Escherichia coli* 0111:B4) was purchased from Sigma-Aldrich Inc. (Saint Louis, MO, USA). Fetal bovine serum (FBS), penicillin–streptomycin, and DMEM culture medium were purchased from Gibco (Pittsburgh, PA, USA). The NO detection kit was purchased from Biyuntian Biological Co., Ltd. (Shanghai, China). Other chemicals were of analytical grade and purchased from Sangon Biotechnology (Shanghai, China).

### 2.2. Sample Collection

Samples were collected from the National Botanical Garden (Beijing, China), and 10- to 14-day-old drone larvae were selected as study subjects. The samples were separated, frozen, and kept at −80 °C for subsequent experiments.

### 2.3. Sample Preparation

The samples were separated into four sections, each of which was dried to a constant weight at different temperatures (80 °C, 50 °C, 30 °C, and −20 °C). The dried samples were ground into powder for later use.

Total lipids were extracted using the modified method of Bligh and Dyer [25]. A total of 40 mL of drone larva powder were placed in a 5 mL glass tube, 300 μL of methanol was added, and the mixture was homogenized. Then, 250 μL of water was added for layering after 600 μL of dichloromethane was added and vortex-mixed three times. After sufficient extraction, the mixture was centrifuged for 15 min at 8000 rpm, and then the lower organic phase was removed into a new glass tube of the same size. All of the aforementioned procedures were completed at ambient temperature. The organic solvent was protected by nitrogen throughout the blow-drying process and was blown dry to the shape of a transparent film. The dried samples were kept in a −80 °C freezer until analysis.

Fatty acids were extracted using the following methods [26]: 2 mL of 0.4 mol/L NaOH-CH_3_OH was added to the 50 mg sample; the mixture was vortexed for 30 s; and the mixture was left at room temperature for 30 min. After vortexing with 2 mL of hexane for 30 s, the hexane phase was transferred to a new centrifuge tube. Two milliliters of hexane were added and vortexed for 30 s. The hexane phase was transferred to the centrifuge tube in the previous step (the hexane phase contains derivatized fatty acid methyl esters). The extracted hexane phase was blown dry under a nitrogen atmosphere. Two milliliters of 5% H_2_SO_4_–CH_3_OH were added to the residual phase, and the reaction was carried out in a water bath at 70 °C for 30 min. Then, 2 mL hexane was added, and the mixture was vortexed for 30 s. In the preceding step, the hexane phase was collected twice and then transferred to a centrifuge tube. After being blown dry, the extracted hexane phase was ultimately redissolved in 100 μL of hexane.

### 2.4. Instrument Conditions and Methods

#### 2.4.1. LC-MS Analysis

Lipid analysis was performed on UPLC-Q-Exactive-Orbitrap–MS (Thermo, San Jose, CA, USA). The detailed mass spectrometer parameters were as follows: spray voltage, 3.2 kV for positive mode and 2.8 kV for negative mode; capillary temperature, 320 °C; aux gas flow rate (arb), 10; mass range (*m*/*z*), 240–2000 for positive mode and 200–2000 for negative mode; full MS resolution, 70,000; MS/MS resolution, 17,500; topN, 10; NCE, 15/30/45; and duty cycle, 1.2 s.

The mobile phase gradient parameters were based on the method of Xu [27]. A Cortecs C18 column (2.1 mm × 100 mm, Waters) was used for LC separation utilizing reversed-phase chromatography. A total of 600 mL of HPLC-grade acetonitrile and 400 mL of HPLC-grade water containing 1 mmol of ammonium acetate were combined for mobile phase A. Mobile phase B contained 10% ACN and 90% IPA (*v*/*v*). Data were acquired using a Q-Exactive-orbitrap mass spectrometer (Thermo, San Jose, CA, USA) coupled with an Ultimate 3000 UHPLC system (Thermo, San Jose, CA, USA). The gradient was as follows: Mobile phase B was maintained at 37% from 0 to 1.5 min; from 1.5 to 4 min, mobile phase B increased to 45%; the percentage of B increased to 52% from 4 to 5 min; and from 5 to 8 min, mobile phase B increased to 58%. At 11 min, mobile phase B increased to 66%; at 11–14 min, mobile phase B increased to 70%; and reached 75% at 18 min. Mobile phase B was increased to 98% at 20 min and stayed there for the next 20 to 22 min. During 22–22.1 min, mobile phase B decreased to 37% and stayed there until 25 min.

#### 2.4.2. GC-MS Analysis

Lipid analysis was performed on GC–MS QP2010plus (SHIMADZU, Kyoto, Japan), which refers to a previous study [28]. A DB-FastFAME (30 m × 0.25 mm, 0.25 μm) column was used. The carrier gas flow rate was maintained at 1.0 mL/min. The heating procedure was as follows: the temperature was maintained at 70 °C for 3 min, and the temperature was raised to 195 °C at a rate of 35 °C/min for 10 min and then to 235 °C at a rate of 10 °C/min for 6 min. The injection port temperature was 250 °C. The sample was injected with a split ratio of 75. The acquisition time ranged from 2 to 26 min. The scan mode was adopted, and the range was 40–500 *m*/*z*. (See Supplementary Table S1 for method validation details).

### 2.5. Cell Culture

Lipopolysaccharide (LPS) can induce the production of proinflammatory cytokines in mouse macrophages (RAW264.7), which is one of the most commonly used in vitro models for inflammation studies [29]. RAW264.7 cell line was cultured in DMEM containing 10% FBS at 37 °C in a 5% CO_2_ moist atmosphere. A 50 mg/mL stock solution of DLL (ultrasound-assisted, dissolved in absolute ethanol) was stored at −20 °C. For use, the stock solution was diluted to various concentrations in DMEM and prepared on the spot. After pretreatment with the indicated concentrations of DLL for 1.5 h, cells were stimulated with LPS (1 μg/mL) in a culture medium at different time points.

### 2.6. Cell Viability Assay

RAW264.7 cells were incubated at 1 × 10^5^ cells/well in 96-well plates for 24 h and then treated with serial concentrations of DLL (5, 20, or 50 μg/mL) for 24 h. Finally, 10 μL of Cell Counting Kit-8 (CCK-8 kit, Dojido, Kumamoto, Japan) was added to each well. After incubation for another 1 h at 37 °C, the absorbance was detected at a wavelength of 450 nm using a microplate reader (Bio-Rad, Model 550, Hercules, CA, USA).

### 2.7. Determination of Nitric Oxide (NO) Concentration in Cell Culture (Greiss Reaction)

RAW264.7 macrophages were incubated in 96-well culture plates and treated with different concentrations of DLL. After 1.5 h of treatment, the cells were stimulated with 1 μg/mL LPS and then incubated for 24 h and 48 h. Cell culture medium was collected for subsequent assays. The concentration of nitric oxide (NO) in the cell culture media was detected using NO kits (Beyotime, Nanjing, China) and measured at 540 nm using a microplate reader (Bio-Rad, model 550, Hercules, CA, USA).

### 2.8. Total RNA Isolation and Quantitative Real-Time PCR

After 24 h of incubation in 12-well plates, RAW264.7 macrophages were exposed to various doses of DLL (5, 20, or 50 μg/mL) for 1.5 h and then stimulated with LPS (1 μg/mL) for 6 h. Using commercial RNA extraction kits (Aidlab Biotechnologies Co., Ltd., Beijing, China) and the PrimeScript RT reagent kit (TaKaRa, Dalian, China), total RNA was extracted and converted to cDNA.

The following primer sequences were used for quantitative real-time PCR: tumor necrosis factor-α (TNF-α) (sense: CCACGCTCTTCTGTCTACTG, antisense: ACTTGGTGGTTTGCTACGAC), inducible nitric oxide synthase (iNOS) (sense: TTTCCAGAAGCAGAATGTGACC, antisense: AACACCACTTTCACCAAGACTC), interleukin-6 (IL-6) (sense: CTCTGCAAGAGACTTCCATCC, antisense: GAATTGCCATTGCACAACTC), interleukin-10 (IL-10) (sense: CTATGCTGCCTGCTCTTACTG, antisense: CAACCCAAGTAACCCTTAAAGTC), cyclooxygenase-2 (COX-2) (sense: GAAATATCAGGTCATTGGTGGAG, antisense: GTTTGGAATAGTTGCTCATCAC), and β-actin (sense: GAGACCTTCAACACCCCAGC, antisense: ATGTCACGCACGATTTCCC). TB Green fluoresced after binding to double-stranded DNA, so it could be used to detect the amplification of PCR products by detecting the fluorescence intensity of TB Green in the reaction system. Quantitative real-time PCR was performed with SYBR premix EX Taq (TaKaRa) according to the standard instructions for the two-step reaction. The 2^−ΔΔCt^ method was used to analyze data for relative gene expression.

### 2.9. Capture of Intracellular ROS Generation

ROS production was determined by a 2,7-dichlorodi-hydrofluorescein diacetate (DCFH-DA) staining assay. In serum-free DMEM, DCFH-DA was diluted 1:1000 to a final concentration of 10 μmol/L. Cell slides were placed in advance in six-well plates, and RAW264.7 macrophages were incubated in six-well plates for 24 h. The experimental groups had a 1.5 h pretreatment with various concentrations of DLL before being exposed to LPS for 24 h. After aspiration of the medium, diluted DCFH-DA was added and incubated in a cell incubator at 37 °C for 20 min to allow adequate binding of DCFH-DA and cells. To completely remove DCFH-DA that had not entered the cells, cell slides were washed twice with serum-free cell culture media DMEM (medium was aspirated after 20 min of immersion) and twice with PBS. Then, cell slides were collected, observed, and photographed. The images were obtained by a Leica TCS SP8 laser scanning confocal microscopy (Leica Microsystem, Wetzlar, Germany). ImageJ.JS software was used to quantify the relative fluorescence intensity.

### 2.10. Statistical Analysis

The Agilent MassHunter workstation carried out mass molecular feature extraction, peak alignment, peak area integral, etc. For statistical analysis, the datasets were subsequently loaded into SIMCA 14.1 software (Sartorius Stedim Biotech Ltd., Ume, Sweden). The statistical significance of variations was examined using SPSS version 26.0 software and one-way analysis of variance (ANOVA). A probability value of *p* < 0.05 was considered statistically significant, and the data from all measurements were expressed as the mean ± standard deviation.

## 3. Results and Discussion

### 3.1. Lipid Composition of DLL

#### 3.1.1. Untargeted Lipidomics Analysis

**Figure 1:**
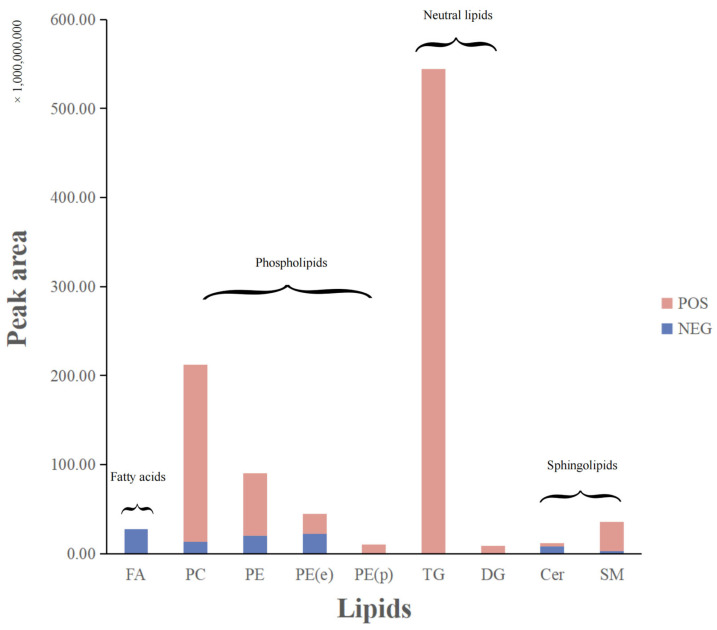
Major lipids were detected in positive and negative ion modes (take the mean of the data from the −20 °C group as an example). According to the ionization mode used to detect lipids, the lipids in the graph are labeled and colored, with the positive mode in red and the negative mode in blue. Cer, ceramide; DG, diacylglycerol; FA, fatty acid; PC, phosphatidylcholine; PE, phosphatidylethanolamine; PE (p), alkyl/alkenyl ether (plasmalogen) phosphatidylethanolamine; SM, sphingomyelin; TG, triacylglycerol.

To ascertain whether temperature conditions had any effect on the quantity and type of lipid compounds in drone larvae, we conducted a comparison study where drone larvae from the same colony aged 10 to 14 days were used. UPLC-Q-Exactive-Orbitrap–MS was used for untargeted analysis of the lipid contents of drone larvae dried under four temperature conditions, combined with the mass spectra, and identified in light of the characteristic ion fragments of lipid substances reported in the literature and databases. In recent years, lipidomics profiling techniques have been widely used to recognize the differences and similarities of lipid substances in biological samples. Agilent Lipid Annotator software (Agilent Profinder B.08.00 software, IDBrowser B.08.00, and Mass Profiler Professional software 15.0) was the main tool to compare the significant elements between samples. The method was proven to be precise, accurate, and sensitive relative to nontargeted analysis. To ensure comparability, we analyzed the same batch of drone larvae samples collected from the same hive, and a total of 21 kinds of lipids were identified, divided into five classes, namely, nine phospholipids, three sphingolipids, two neutral lipids, one plant-based glycoglycerolipid, four fatty acyls, and other lipids. Among them, the major components of phospholipids were CL (18:1), PC (18:1), PE (18:1), PG (18:1), and PI (18:1) (Supplementary Table S1). PC, PE, PE (e), PE (p), TG, DG, and SM were most frequently found in the positive ion mode, while the negative ion mode revealed the presence of FA, PC, PE (e), PE (p), and Cer (Figure 1). The results presented above are in line with those demonstrated in previous research [30,31].

**Figure 2:**
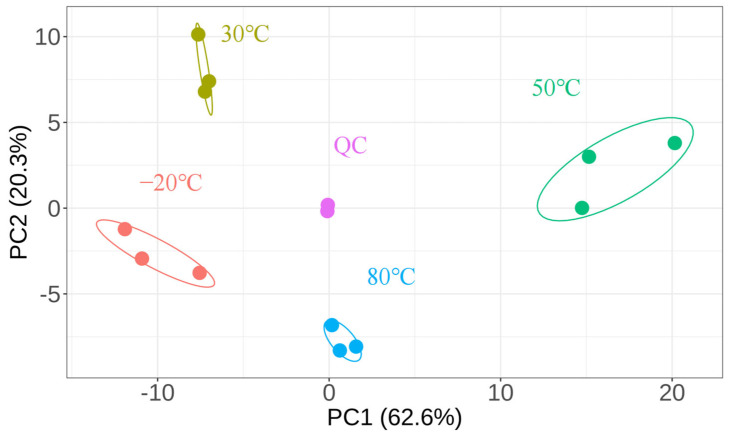
PCA score plots of lipids in the samples under different drying conditions.

To visualize the overall differences between the samples, PCA was performed on the tested samples (Figure 2). The data points of the same sample were distributed centrally, with less dispersion and overlap. A total of 82.9% of the overall variation was explained by the two-dimensional PCA. The overall dataset variability was explained by the first principal component (PC1) at 62.6% and the second principal component at 20.3%. The four samples’ distribution zones were obviously distinct from one another. The distribution of the 80 °C and 50 °C samples was predominantly on the PC1 positive axis, whereas the distribution of the 30 °C and −20 °C samples was predominantly on the PC1 negative axis. The fact that the quality control group (QC) did not scatter suggests the stability of the experimental set. PCA showed that the four groups of samples were clearly distinguished, indicating that changing the drying conditions led to differences in the content of lipid compounds, supporting the role of temperature in aiding the distribution of the lipid composition.

**Figure 3:**
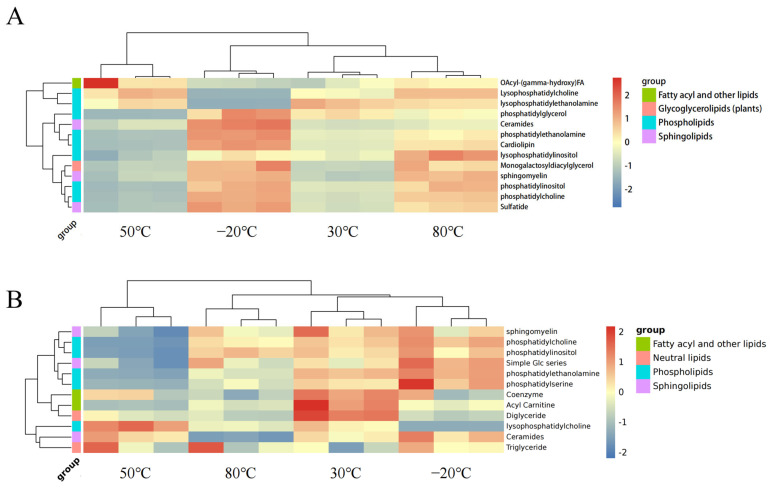
Cluster analysis heatmap of lipid substances extracted from drone larvae under different drying conditions. Red and blue represent high and low expression levels of metabolites, respectively. (**A**) negative ion mode; (**B**) positive ion mode.

For a more comprehensive investigation of the data, a clustered heatmap was used to analyze the lipid compounds (Figure 3). Each tiny square represents a particular lipid, and the color of each square shows the amount of that element. Red denotes upregulation, while blue indicates downregulation. It is clear that there are considerable changes in lipid components and that there are major variances between the various temperature groups. We found that the samples from the −20 °C group contained a higher level of Acyl-(gamma-hydroxy) FA compared to the other groups. Thus, in the subsequent experiments, we performed further quantitative analysis of fatty acids in the samples of the four groups with the help of GC-MS.

As observed, 80 °C samples had considerably lower levels of phosphatidylethanolamine, fatty acids, and ceramides, as well as less Acyl-(gamma-hydroxy) FA than 30 °C samples and −20 °C samples. For the 50 °C samples, the quantities of cardiolipin, phosphatidylinositol, phosphatidylethanolamine, and ceramides were also drastically reduced. This suggests that the variation in temperature might lead to the redistribution of several lipid elements.

#### 3.1.2. Free Fatty Acid (FFA) Analysis

To better comprehend the variations between the four groups of drone larvae, we examined the primary active fatty acids of the samples. Further targeted quantitative analysis of fatty acids was performed by GC–MS, and 18 fatty acids out of a total of 37 were detected in the samples (Table 1), including, interestingly, caprylic, capric, lauric, tridecanoic, myristic, myristoleic, pentadecanoic, palmitic, palmitoleic, margaric, stearic, oleic, linoleic, linolenic, arachidic, cis-5-eicosenic, behenic, and lignoceric acids.

The samples’ predominant fatty acids at the four different temperatures were myristic (240.79 ± 17.26 μg/g), palmitic (1662.64 ± 80.31 μg/g), stearic (827.21 ± 50.85 μg/g), and oleic (2363.65 ± 168.11 μg/g) acids. When compared to the 50 °C samples, the contents of capric acid and myristoleic acid in the 80 °C samples were significantly higher (*p* < 0.05). In addition, the palmitic and margaric acid levels in the 50 °C samples were considerably lower than those in the −20 °C samples and 30 °C samples (*p* < 0.05), while the lauric acid content in the 80 °C samples and −20 °C samples was significantly higher than that in the 50 °C samples (*p* < 0.001 and *p* < 0.05). In comparison to other groups, the palmitoleic content in the 50 °C samples was significantly lower (*p* < 0.05). The content of margaric acid in the 80 °C samples was significantly lower (*p* < 0.05) than that in the −20 °C samples and 30 °C samples. In addition to the fatty acid differences described above, tridecanoic acid was detected only in 30 °C samples. In addition, linoleic, linolenic, and other polyunsaturated fatty acids (Supplementary Table S2) were also found in the samples. There is a large amount of literature and experimental evidence demonstrating that lipids have anti-inflammatory properties [32,33,34]. However, there have been few lipidomics studies targeting the anti-inflammatory efficacy of DLLs. As a consequence of the previously mentioned findings, we chose the DLLs produced after freeze-drying at −20 °C as the experimental object. We conducted in vitro anti-inflammatory tests to assess DLL’s anti-inflammatory activity in the subsequent investigations.

### 3.2. In Vitro Anti-Inflammatory Activity of DLL

#### 3.2.1. Effect of DLL on Cell Viability

Before examining DLL’s anti-inflammatory functions, the possible cytotoxicity of DLL in RAW264.7 cells was investigated using the CCK-8 assay (Supplementary Figure S1). As shown in Supplementary Figure S1, 5 μg/mL DLL significantly increased cell viability (*p* < 0.001), whereas 1000 μg/mL DLL significantly inhibited cell growth (*p* < 0.005). When the concentration of DLL was below 100 μg/mL, the cell viability was higher than that of the control group. The data presented above validate the adjustment of the DLL concentration range in future research.

#### 3.2.2. DLL Inhibited the Production of NO in LPS-Activated RAW264.7 Macrophages

**Figure 4:**
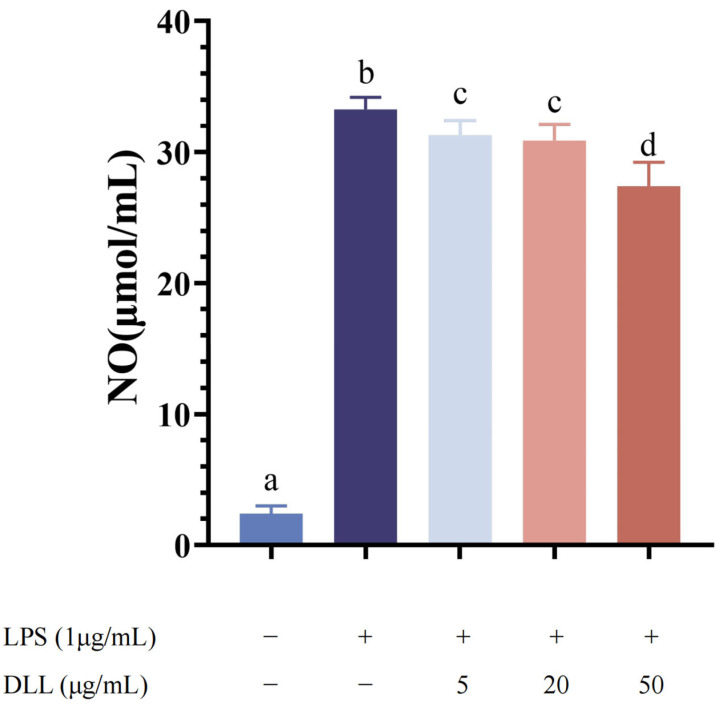
Effect of DLL on NO production in RAW264.7 cells with LPS-induced inflammation. RAW264.7 cells were pretreated with the indicated concentrations of three kinds of BPL for 1.5 h and then stimulated with LPS (1 μg/mL) for 24 h. The LPS group was obtained in the absence of DLL. The control group was obtained in the absence of LPS and DLL. Different letters indicate significant differences between groups (*p* < 0.05).

The data were verified by reflecting the concentration of NO through the color of the reagent after the color reaction. As a result, a standard curve of NO concentration and absorbance was established, which had a linear range of 0–100 μM, with a formula of y = 0.0044x + 0.0482 and an R^2^ value of 0.9987. As seen in Figure 4, very low NO concentrations were found after 24 h of incubation without LPS. The release of NO was greatly increased in the LPS group (*p* < 0.001), demonstrating that LPS (1 μg/mL) could significantly boost NO release. However, the production of NO was considerably and dose-dependently reduced after 1.5 h of DLL pretreatment. The inhibition of NO production was strongest when the concentration of DLL was 50 μg/mL (*p* < 0.0001).

#### 3.2.3. DLLs Inhibited LPS-Induced IL-6, IL-10, COX-2, and iNOS mRNA Expression in RAW264.7 Macrophages

**Figure 5:**
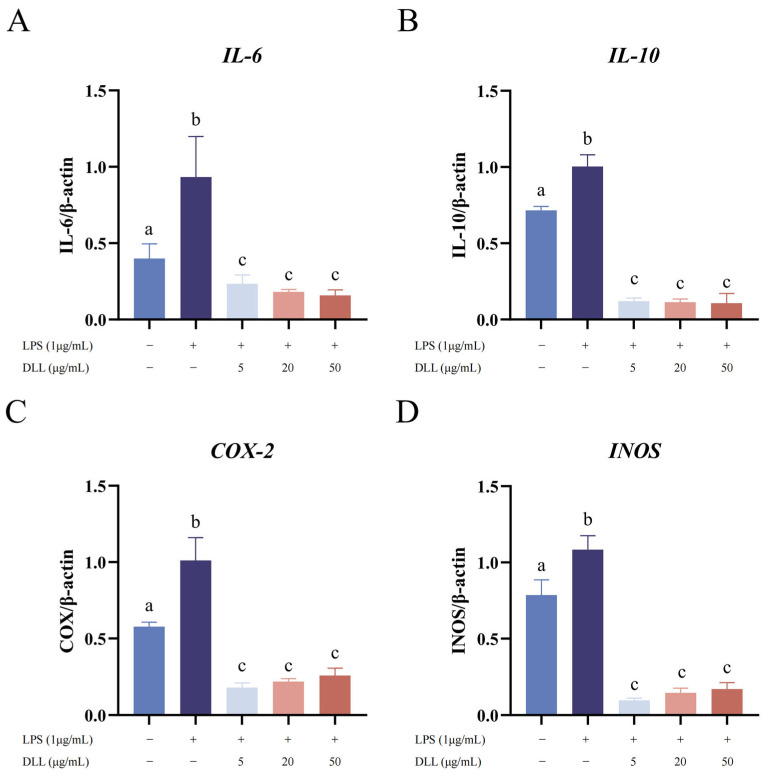
Effect of DLL on LPS-stimulated RAW264.7 cell mRNA expression of important inflammatory mediators and cytokine genes, including IL-6 (**A**), IL-10 (**B**), COX-2 (**C**), and iNOS (**D**). RAW264.7 cells were pretreated with DLL at the designated concentrations for 1.5 h and then stimulated with LPS (1 μg/mL) for 6 h. The LPS group was obtained in the absence of DLL. A control check group was obtained in the absence of LPS and DLL. Different letters indicate significant differences between groups (*p* < 0.05).

**Figure 6:**
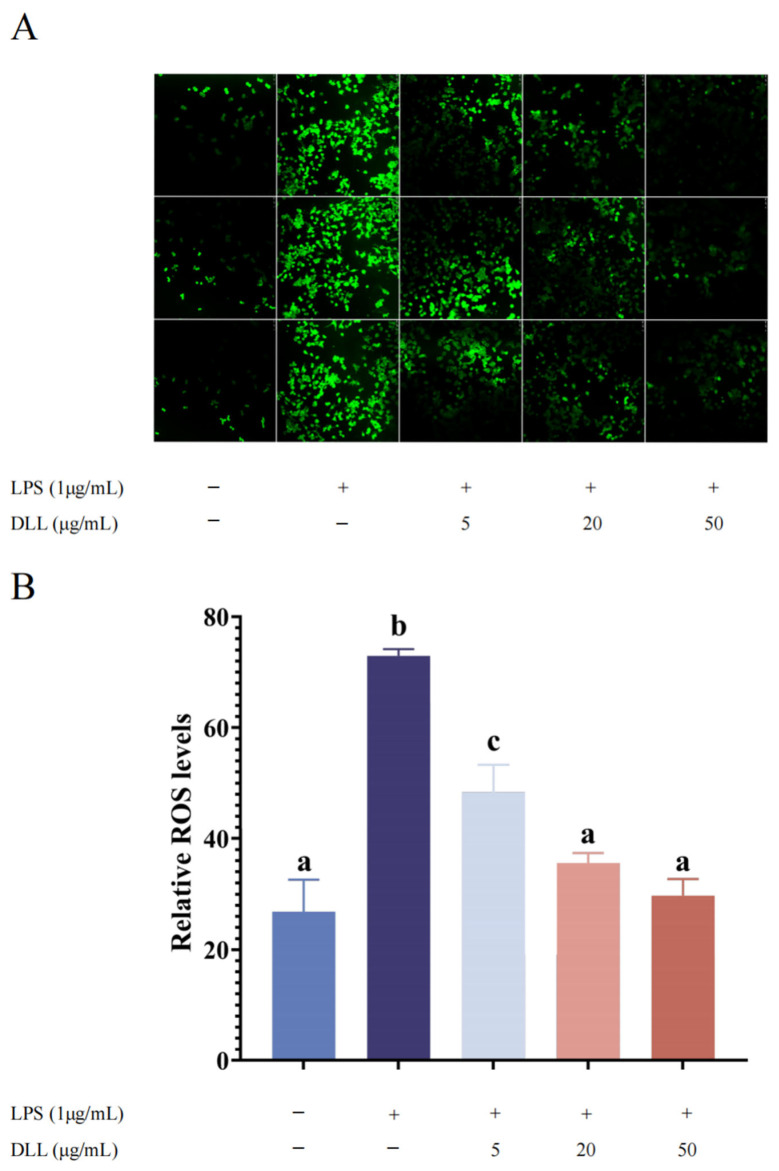
Effects of different concentrations of DLL on the ROS level of RAW264.7 cell. RAW264.7 cells were pretreated with DLL (n = 3) for 1.5 h before LPS (1 μg/mL) stimulation for another 24 h. (**A**) ROS was captured by a Leica TCS SP8 laser scanning confocal microscope with 10 μM DCFH-DA diacetate (2′,7′-Dichlorodihydrofluorescein diacetate). (**B**) Relative fluorescence quantification of ROS based on ImageJ software. Different letters indicate significant differences between groups (*p* < 0.05).

iNOS is a high-output Ca++-independent NOS whose gene expression can be induced in a wide range of cells and tissues by cytokines and other agents [35]. The results showed that the mRNA expression level of iNOS was upregulated in RAW264.7 macrophages stimulated by LPS (1 μg/mL) (Figure 5). However, DLL treatment significantly reduced the level of iNOS in a dose-dependent manner compared with LPS stimulation (*p* < 0.0001). This result is consistent with previous findings that showed a decrease in NO production in association with the inhibition of iNOS expression. In addition, as indicated in Figure 6, IL-6, IL-10, and COX-2 were among the inflammatory proteins whose expression was dramatically upregulated by LPS stimulation. However, the expression of these inflammatory factors was considerably and dose-dependently reduced after DLL administration (*p* < 0.0001).

NO is a signaling molecule that plays a key role in the pathogenesis of inflammation. With the help of NOSs, NO is produced via the conversion of arginine to citrulline, which then releases NO into endothelial cells [36]. On the one hand, NO has anti-inflammatory effects under normal physiological conditions. On the other hand, NO is regarded as a proinflammatory mediator since it produces excessive NO under unusual conditions, triggering the inflammatory cascade [37,38,39]. Therefore, the inhibition of NO secretion can be used to evaluate whether chemical components have anti-inflammatory effects. In addition, the inhibition of INOS mRNA expression is one of the key indicators of anti-inflammation since INOS is the main regulator of NO generation in RAW264.7 cells [22]. In addition, IL-6 can stimulate the proliferation of activated B cells and secrete antibodies [40]. IL-10 plays a role in the suppression of the inflammatory response and in antagonizing inflammatory mediators [41]. COX-2 is an immediate, early-response gene that is highly inducible by inflammatory stimuli [42]. Therefore, inhibition of the mRNA expression of these three genes could also represent an inhibitory pathway.

After LPS (1 μg/mL) administration, the in vitro inflammatory cell response model was effectively established, as evidenced by the dramatically increased NO release (Figure 4) and significantly elevated mRNA expression of IL-6, IL-10, COX-2, and iNOS (Figure 5). Compared to the LPS stimulation group, DLL therapy dramatically reduced the mRNA expression of iNOS, IL-6, IL-10, and COX-2 in a dose-dependent pattern. Additionally, the results of the CCK-8 test revealed that DLL at concentrations below 1000 g/mL did not significantly inhibit cell viability (Supplementary Figure S1), indicating that DLL treatment had an anti-inflammatory impact and could successfully combat inflammation.

#### 3.2.4. DLLs Inhibited LPS-Induced ROS Level in RAW264.7 Macrophages

Overproduction of ROS causes oxidative stress, which is a key factor in the inflammatory process in cells [43,44]. We evaluated the effect of DLL on ROS production in LPS-induced macrophage RAW264.7 cells to further investigate its protective function. Quantification of relative ROS fluorescence based on ImageJ showed that the fluorescence intensity of the blank control group was 26.83 ± 5.79, and the relative fluorescence intensity of the LPS group was 72.95 ± 1.20, indicating that LPS induction increased the intracellular ROS levels in RAW264.7 cells (*p* < 0.001). The relative fluorescence intensity decreased to 48.37 ± 4.94, 35.56 ± 1.81, and 29.74 ± 2.97 after pretreatment with 5, 20, and 50 (μg/mL) concentrations of DLL, respectively. Compared with LPS-stimulated model cells, pretreatment with DLL for 1.5 h significantly reduced LPS-induced ROS production in a dose-dependent manner (Figure 6), suggesting that DLL protects macrophages from LPS-induced oxidative stress. This is consistent with prior results showing that PUFA can inhibit ROS generation in HAECs [45]. As a consequence, we propose that DLL exerts a protective effect against LPS-induced inflammation by regulating oxidative stress.

These results conclusively show that lipids extracted from drone larvae have anti-inflammatory properties, as supported by several studies [46,47,48,49]. Thus, we proposed that DLL may likewise be applied to treat inflammatory diseases in vivo.

## 4. Conclusions

In summary, we performed a comprehensive lipidomic analysis of drone larvae based on the UPLC-Q-Exactive Orbitrap/MS method. DLLs extracted from drone larvae (dried at −20 °C, 30 °C, 50 °C, and 80 °C) were examined. The outcome was displayed as a change in the lipid components and was characterized by combining targeted and untargeted analyses, potentially providing a reference for insect food quality management. Furthermore, −20 °C-derived DLL displayed in vitro anti-inflammatory activities in LPS-stimulated RAW264.7 cells by suppressing NO and ROS generation and decreasing the mRNA expression of IL-6, IL-10, COX-2, and iNOS. In addition, DLL was found to possess antioxidant properties, which warrants further investigation. The findings suggest that integrating drones as a potential raw material for dietary supplements presents exciting possibilities, yet further research is needed to determine their safety and efficacy. This study highlights an opportunity for beekeepers to potentially diversify their product offerings and drives future investigations into the potential benefits of drone larvae.

## Figures and Tables

**Table 1:** Composition and content of fatty acids in drone larvae.

Fatty Acid	RT (min)	Content (μg/g)			
		−20 °C	30 °C	50 °C	80 °C
Caprylic	5.153	0.13 ± 0.02	0.12 ± 0.02	0.14 ± 0.02	0.16 ± 0.01
Capric	6.111	1.52 ± 0.12 ^a,b^	1.40 ± 0.15 ^a,b^	1.30 ± 0.13 ^a^	1.67 ± 0.03 ^b^
Lauric	6.832	2.67 ± 1.89 ^a,c^	2.56 ± 1.17 ^a,b,c^	2.27 ± 1.96 ^b,c^	2.85 ± 0.29 ^a,c^
Tridecanoic	7.179	\	0.07 ± 0.1	\	\
Myristic	7.552	246.99 ± 13.19 ^a^	250.21 ± 5.51 ^a^	216.32 ± 14.37 ^b^	249.66 ± 4.58 ^a^
Myristoleic	7.722	2.10 ± 0.04 ^a,b^	2.25 ± 0.29 ^a,b^	1.76 ± 0.19 ^a^	2.33 ± 0.25 ^b^
Pentadecanoic	7.98	0.76 ± 0.04	0.69 ± 0.04	0.68 ± 0.10	0.74 ± 0.02
Palmitic	8.494	1707.87 ± 60.53 ^a,b^	1721.35 ± 12.41 ^a,b^	1553.33 ± 69.13 ^c^	1668.02 ± 25.92 ^a,b,c^
Palmitoleic	8.684	52.51 ± 4.56 ^a^	53.52 ± 3.43 ^a^	42.51 ± 2.07 ^b^	51.78 ± 2.07 ^a^
Margaric	9.133	2.89 ± 0.14 ^a,b^	2.83 ± 0.10 ^a,b^	2.59 ± 0.19 ^a^	3.17 ± 0.10 ^b^
Stearic	9.946	852.32 ± 24.17	844.87 ± 44.72	772.05 ± 56.99	839.63 ± 46.73
Oleic	10.192	2463.03 ± 149.61	2363.70 ± 198.71	2189.96 ± 134.92	2437.91 ± 83.85
Linoleic	10.71	41.84 ± 0.39	41.88 ± 5.68	36.03 ± 3.22	43.43 ± 3.07
Linolenic	11.452	44.35 ± 1.73	47.24 ± 2.37	34.66 ± 1.79	45.08 ± 3.87
Arachidic	12.39	28.76 ± 0.72	28.56 ± 1.49	29.32 ± 2.87	32.42 ± 1.28
Eicosenic cis 5	12.806	7.00 ± 0.56	6.41 ± 0.34	6.20 ± 0.54	5.81 ± 0.36
Behenic	16.631	3.89 ± 0.10	3.89 ± 0.32	7.72 ± 0.85	5.07 ± 0.81
Lignoceric	19.989	3.19 ± 0.25	2.95 ± 0.61	37.21 ± 16.11	5.71 ± 1.21

Values are the mean ± standard deviation (n = 3). Means in a row with different letters differ at *p* < 0.05. “\” means not detected.

## Data Availability

The data used to support the findings of this study can be made available by the corresponding author upon request.

## Supplementary Materials

The following supporting information can be downloaded at: https://www.mdpi.com/article/10.3390/foods12224058/s1, Table S1: LOD, LOQ, R2, linear formula, and linear range of standard of 37 fatty acid methyl esters. Table S2: FA analysis percentage of DLL. Figure S1: Effects of DLL on the cell viability of RAW 264.7 cells. RAW 264.7 cells were treated with the indicated concentrations of DLL for 24 h. Different letters indicate significant differences between groups (*p* < 0.05). Figure S2: Results of identification of drone larvae. (A) Gel electrophoresis of the mitochondrial (16S) rRNA genes of *Apis mellifera*. (B) Neihbor Jioning tree among drone larvae, *Apis mellifera.ligustica*, *Apis mellifera.caucasica*, *Apis koschevnikovi*, *Apis dorsata*, *Apis nuluensis*, *Apis cerana* and *Bombus brevivillus*. Figure S3: Results of authenticity verification of RAW 264.7 cell lines. Eighteen short tandem repeat (STR) loci were amplified using multiplex PCR. One additional marker (Human TH01) was used to screen for the presence of human species. The cell line sample was processed using the ABI Prism^®^ 3130 XL Genetic Analyzer. Data were analyzed using Gene Mapper^®^ ID v3.2 software (Applied Biosystems). Appropriate positive and negative controls were run and confirmed for each sample submitted. Cell lines were authenticated using Short Tandem Repeat (STR) analysis as described in Almeida J.L. et al. [50].
