# Supplementary material for: Total Lipid Extracts of Honeybee Drone Larvae Are Modulated by Extraction Temperature and Display Consistent Anti-Inflammatory Potential
Source: Foods. 2023 Nov 8;12(22):4058. doi: 10.3390/foods12224058 (PMC10670194; doi:10.3390/foods12224058)
Supplement: Supplementary file 1 [file foods-12-04058-s001.zip › foods-2580555-supplementary.pdf]

Table S1. LOD, LOQ, R2, linear formula, and linear range of standard of 37 fatty acid methyl esters.

Number	Fatty acid	LOQ mg/L	LOD mg/L	R^2	Formula	Linear range mg/L
1	C4:0	0.1	0.25	0.9997456	Y = 3036.299X + 1566.217	0.25-65.75
2	C6:0	0.1	0.25	0.9997885	Y = 7131.883X + 1873.404	0.25-65.75
3	C8:0	0.1	0.25	0.9999602	Y = 1008.449X + 132.3901	0.25-65.75
4	C10:0	0.1	0.25	0.9999553	Y = 5430.702X + 165.7051	0.25-65.75
5	C11:0	0.1	0.25	0.9999865	Y = 12114.43X - 747.8309	0.25-65.75
6	C12:0	0.1	0.25	0.9999855	Y = 12596.27X - 1033.914	0.25-65.75
7	C13:0	0.1	0.25	0.9999794	Y = 12916.48X - 1566.949	0.25-65.75
8	C14:0	0.1	0.25	0.9999568	Y = 12934.32X - 2630.947	0.25-65.75
9	C14:1	0.1	0.25	0.9995251	Y = 3014.028X - 1088.863	0.25-65.75
10	C15:0	0.1	0.25	0.9999114	Y = 12976.97X - 3721.518	0.25-65.75
11	C15:1	0.1	0.25	0.9998915	Y = 5826.711X - 1496.289	0.25-65.75
12	C16:0	0.1	0.25	0.9999516	Y = 24793.63X - 3303.589	0.25-1000
13	C16:1	0.1	0.25	0.9993959	Y = 2806.22X - 1286.494	0.25-65.75
14	C17:0	0.1	0.25	0.9996139	Y = 12227.62X - 5673.093	0.25-65.75
15	C17:1	0.1	0.25	0.9993035	Y = 2530.643X - 1290.526	0.25-65.75
16	C18:0	0.1	0.25	0.9993899	Y = 11863.58X - 6084.826	0.25-1000
17	C:18:1 trans	0.1	0.25	0.9994892	Y = 4437.022X - 2273.598	0.25-65.75
18	C:18:1 cis	0.1	0.25	0.9996076	Y = 4613.939X - 1802.2	0.25-1000
19	C:18:2 trans	0.1	0.25	0.9992071	Y = 5323.399X - 3287.905	0.25-65.75
20	C:18:2 cis	0.1	0.25	0.9993318	Y = 5288.806X - 2721.397	0.25-1000

21	C:18:3 n6	0.1	0.25	0.9992161	Y = 4562.919X - 2951.752	0.25-65.75
22	C:18:3 n3	0.1	0.25	0.9989127	Y = 5974.833X - 4356.01	0.25-1000
23	C20:0	0.1	0.25	0.9987174	Y = 10631.24X - 8069.17	0.25-65.75
24	C20:1	0.1	0.25	0.9992571	Y = 4555.069X - 3224.338	0.25-65.75
25	C20:2	0.25	0.5	0.9987198	Y = 4813.764X - 4647.128	0.5-65.75
26	C21:0	0.1	0.25	0.9983389	Y = 10672.47X - 8975.879	0.25-65.75
27	C20:3 n6	0.25	0.5	0.9986952	Y = 4513.715X - 4728.395	0.5-65.75
28	C20:4 n6	0.25	0.5	0.9983858	Y = 4542.233X - 5184.685	0.5-65.75
29	C20:3 n3	0.5	1	0.9982756	Y = 5651.922X - 8506.479	1.0-65.75
30	C20:5 n3 EPA	0.5	1	0.9983207	Y = 4802.876X - 6863.904	1.0-65.75
31	C22:0	0.5	1	0.9980549	Y = 10147.78X - 16740.45	1.0-65.75
32	C22:1	0.5	1	0.9990206	Y = 4398.465X - 5539.293	1.0-65.75
33	C22:2 n6	0.25	0.5	0.9969498	Y = 4458.682X - 6070.515	0.25-65.75
34	C23:0	0.1	0.25	0.9973358	Y = 9496.094X - 10393.36	0.25-65.75
35	C24:0	1	2	0.999141	Y = 692.462X - 1320.493	2.0-65.75
36	C22:6 DHA	0.5	1	0.9986089	Y = 4858.014X - 5571.132	1.0-65.75
37	C24:1	0.5	1	0.9989	Y = 4675.496X - 6335.448	1.0-65.75

Table S2. FA analysis percentage of DLL.

Classification		FA analysis percentage (%)			
		20	30	50	80
Cardiolipin (CL)	CL(16:0)	2.52±0.09	2.55±0.07	2.58±0.08	2.57±0.28
	CL(16:1)	1.28±0.05	1.33±0.1	1.25±0.03	1.3±0.01
	CL(18:1)	76.63±0.55	76.19±0.65	77.25±1.08	75.55±1.22
	CL(18:2)	10.16±0.45	10.35±0.47	7.89±0.74	10.52±0.84
	CL(18:3)	1.76±0.07	1.87±0.22	1.47±0.27	2±0.25
	CL(20:4)	7.65±0.13	7.72±0.14	9.57±0.05	8.05±0.17
Lysophosphatidylcholine (LPC)	LPC(16:0)	17.38±0.25	15.55±0.21	10.59±0.23	9.49±0.81
	LPC(18:0)	5.35±0.13	4.63±0.21	2.75±0.3	1.92±0.4
	LPC(18:1)	77.27±0.13	79.82±0.08	86.66±0.49	88.59±1.21
Lysophosphatidylethanolamine (LPE)	LPE(16:0)	7.73±0.13	7.16±0.34	4.9±0.2	3.86±0.59
	LPE(18:0)	26.67±0.29	22.72±0.79	11.24±0.93	4.68±1.03
	LPE(18:1)	65.59±0.18	70.11±0.7	83.86±1.03	91.47±1.61
Lysophosphatidylethanolamine (LPI)	LPI(18:1)	13.79±3.5	18.28±4.67	46.63±2.34	27.31±2.94
	LPI(18:3)	86.21±3.5	81.72±4.67	53.37±2.34	72.69±2.94
Phosphatidylcholine (PC)	PC(14:0)	1.41±0.12	1.37±0.04	0.65±0.05	1.2±0.03
	PC(16:0)	12.11±0.37	12.41±0.13	19.18±0.19	13.46±0.3
	PC(16:1)	2.04±0.1	2.01±0.03	0.83±0.07	1.87±0.05
	PC(18:0)	9.85±0.24	9.74±0.56	9.29±0.31	9.75±0.25
	PC(18:0e)	0.37±0.03	0.4±0.03	0.79±0.07	0.44±0.12
	PC(18:1)	68.86±0.44	68.75±0.36	66.3±0.49	68.46±0.14

Classification		FA analysis percentage (%)			
		20	30	50	80
	PC(18:1e)	0.4±0.04	0.44±0.04	1.01±0.17	0.5±0.21
	PC(18:2)	2.16±0.07	2.13±0.17	0.85±0.04	1.89±0.04
	PC(18:3)	2.11±0.09	2.06±0.12	0.65±0.11	1.82±0.41
	PC(19:0)	0.14±0	0.13±0.01	0.09±0.02	0.12±0.02
	PC(20:0)	0.3±0.02	0.3±0.02	0.22±0	0.26±0.02
	PC(20:1)	0.25±0.02	0.24±0	0.13±0.01	0.23±0.02
Phosphatidylethanolamine (PE)	PE(14:0e)	0.04±0	0.04±0	0.02±0	0.03±0
	PE(16:0)	4.74±0.11	4.5±0.05	2.92±0.13	4.12±0.24
	PE(16:0e)	0.43±0.02	0.41±0.02	0.32±0.03	0.29±0.02
	PE(16:1)	0.18±0.01	0.16±0.01	0.05±0.01	0.11±0.01
	PE(16:1e)	0.18±0.01	0.17±0.01	0.11±0.01	0.12±0
	PE(17:0)	0.07±0	0.06±0	0.02±0	0.05±0
	PE(18:0)	10.11±0.18	9.77±0.15	6.66±0.46	9.69±0.55
	PE(18:0e)	15.89±0.17	16.94±0.16	25.38±0.91	18.7±0.8
	PE(18:1)	54.44±0.24	54.36±0.12	52.58±0.39	55.25±0.12
	PE(18:1e)	6.69±0.27	6.65±0.28	6.84±0.56	6.32±1.5
	PE(18:2)	1.25±0.05	1.18±0.02	0.72±0.05	0.9±0.01
	PE(18:3)	2.24±0.22	2.05±0.08	1.19±0.55	1.4±0.14
	PE(19:0)	0.08±0	0.08±0.01	0.03±0	0.06±0.01
	PE(20:0)	0.35±0.01	0.32±0.01	0.12±0.01	0.24±0
	PE(20:0e)	1.57±0.09	1.58±0.03	1.57±0.03	1.32±0.03

Classification		FA analysis percentage (%)			
		20	30	50	80
	PE(20:1e)	1.62±0.12	1.61±0.02	1.37±0.08	1.3±0.13
	PE(20:2e)	0.11±0	0.11±0	0.1±0.01	0.09±0
Phosphatidylglycerol (PG)	PG(16:0)	9.74±0.11	9.46±0.3	12.12±0.45	10.91±0.85
	PG(18:0)	1.17±0.11	1.25±0.04	3.86±0.8	1.54±0.19
	PG(18:1)	89.09±0.2	89.29±0.34	84.02±1.12	87.55±1.04
Phosphatidylglycerol (PI)	PI(16:0)	15.85±0.28	15.88±0.15	18.26±0.59	16.18±0.24
	PI(18:0)	21.54±0.36	21.49±0.23	19.14±0.44	20.23±1.05
	PI(18:1)	42.73±0.48	42.49±0.74	46.15±0.89	43.07±0.69
	PI(18:2)	6.41±0.19	6.44±0.29	6.08±0.2	6.22±0.31
	PI(18:3)	13.47±0.28	13.7±0.2	10.37±0.77	14.29±0.88
Acyl Carnitine (AcCa)	AcCa(16:0)	24.27±0.82	41.16±3.32	45.86±7.56	26.12±0.7
	AcCa(18:1)	75.73±0.82	58.84±3.32	54.14±7.56	73.88±0.7
Diglyceride (DG)	DG(16:0)	22.24±0.14	12.44±0.14	19.67±0.31	19.36±0.08
	DG(18:0)	3.64±0.07	3.86±0.12	4.73±0.12	3.54±0.24
	DG(18:1)	71.78±0.1	80.37±0.07	72.97±0.26	73.94±0.14
	DG(18:2)	0.82±0.03	1.33±0.02	1.05±0.03	1.31±0.07
	DG(18:3)	1.52±0.11	2±0.17	1.58±0.11	1.85±0.24
Triglyceride (TG)	TG(12:0)	2.35±0.07	2.07±0.06	2.24±0.15	2.24±0.06
	TG(14:0)	6.54±0.09	6.55±0.03	6.21±0.13	6.27±0.08
	TG(14:1)	0.93±0.04	0.83±0.03	0.88±0.05	0.94±0.06
	TG(15:0)	0.44±0.01	0.44±0	0.44±0.03	0.44±0.01

Classification	FA analysis percentage (%)			
	20	30	50	80
TG(16:0)	35.7±0.12	35.79±0.26	35.3±0.07	34.89±0.29
TG(16:1)	2.21±0.01	2.06±0.03	2.13±0.09	2.27±0.05
TG(17:0)	0.45±0.01	0.45±0.01	0.45±0.01	0.46±0
TG(17:1)	0.03±0	0.03±0	0.03±0	0.03±0
TG(18:0)	14.56±0.28	15.17±0.2	15.19±0.19	14.79±0.09
TG(18:1)	31.91±0.27	32.26±0.24	32.34±0.54	32.38±0.23
TG(18:2)	0.35±0.01	0.32±0.01	0.36±0.05	0.4±0.04
TG(18:3)	2.85±0.09	2.73±0.14	2.74±0.28	3.2±0.26
TG(20:0)	0.23±0	0.2±0.02	0.2±0.02	0.23±0
TG(20:1)	0.02±0	0.02±0	0.02±0	0.02±0
TG(22:0)	0.04±0	0.04±0	0.04±0	0.04±0
TG(23:0)	0.03±0	0.03±0	0.03±0	0.03±0
TG(24:0)	0.01±0	0.01±0	0.03±0	0.01±0
TG(25:0)	0.02±0	0.02±0	0.03±0	0.02±0
TG(26:0)	0.04±0	0.03±0.01	0.03±0.01	0.03±0
TG(4:0)	1.24±0.11	0.9±0.08	0.03±0.2	1.23±0.15
TG(8:0)	0.06±0	0.04±0	0.03±0.01	0.06±0.01

Values are mean ± standard deviation (n = 3).

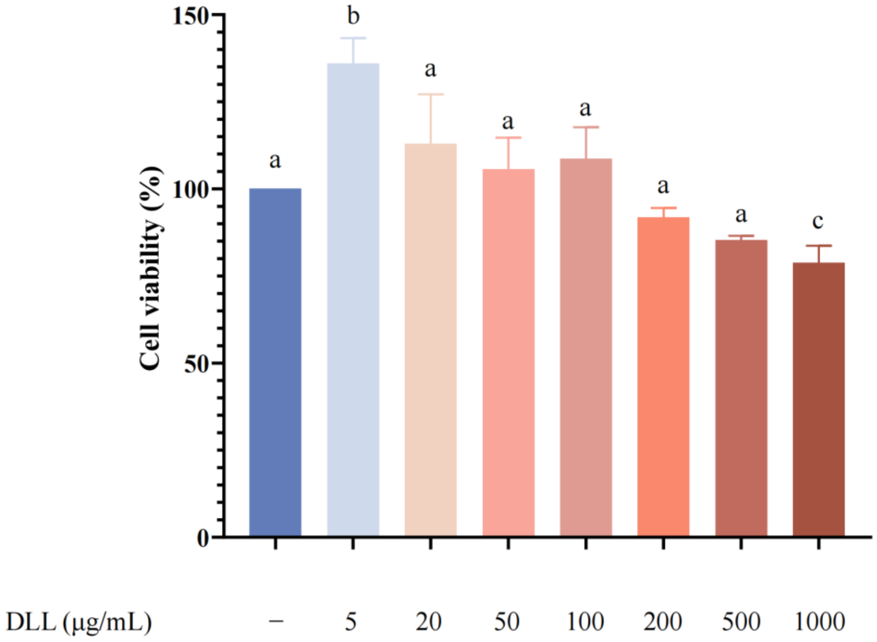

Figure S1. Effects of DLL on the cell viability of RAW 264.7 cells. RAW 264.7 cells were treated with the indicated concentrations of DLL for 24 h. Different letters indicate significant differences between groups ($p < 0.05$).

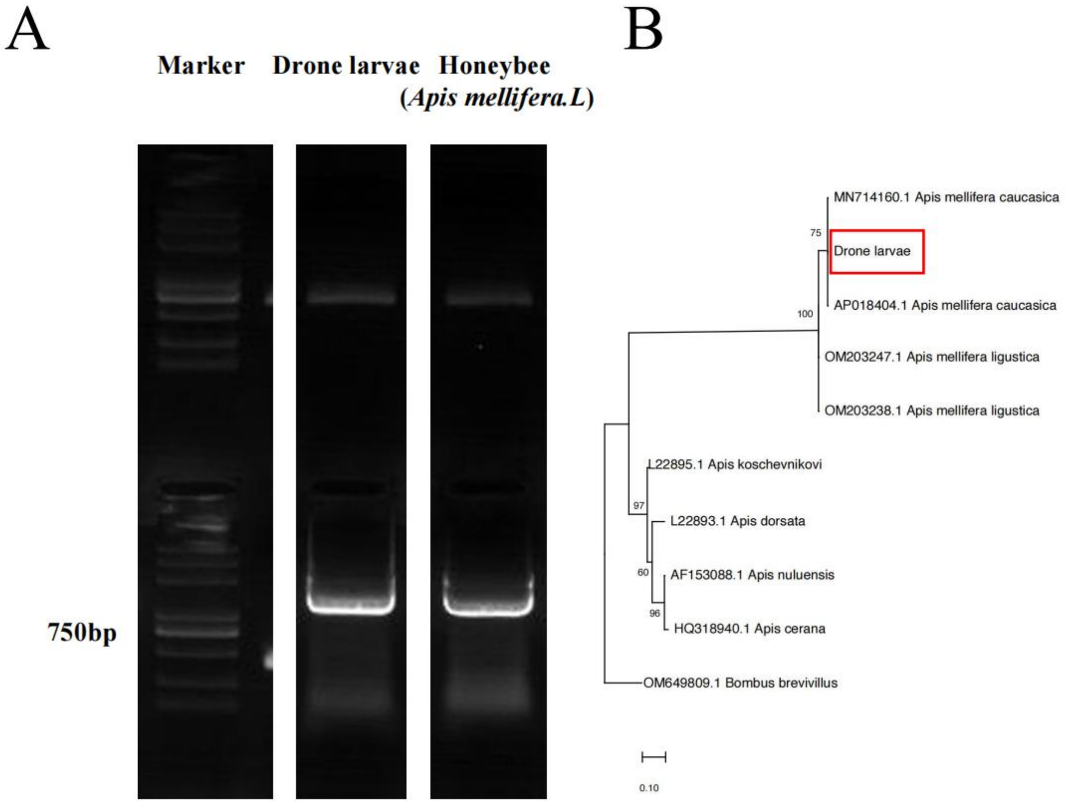

Figure S2. Results of identification of drone larvae. (A) Gel electrophoresis of the mitochondrial (16S) rRNA genes of Apis mellifera. (B)Neihbor Jioning tree among drone larvae, Apis mellifera.ligustica, Apis mellifera.caucasica, Apis koschevnikovi, Apis dorsata, Apis nuluensis, Apis cerana and Bombus brevivillus

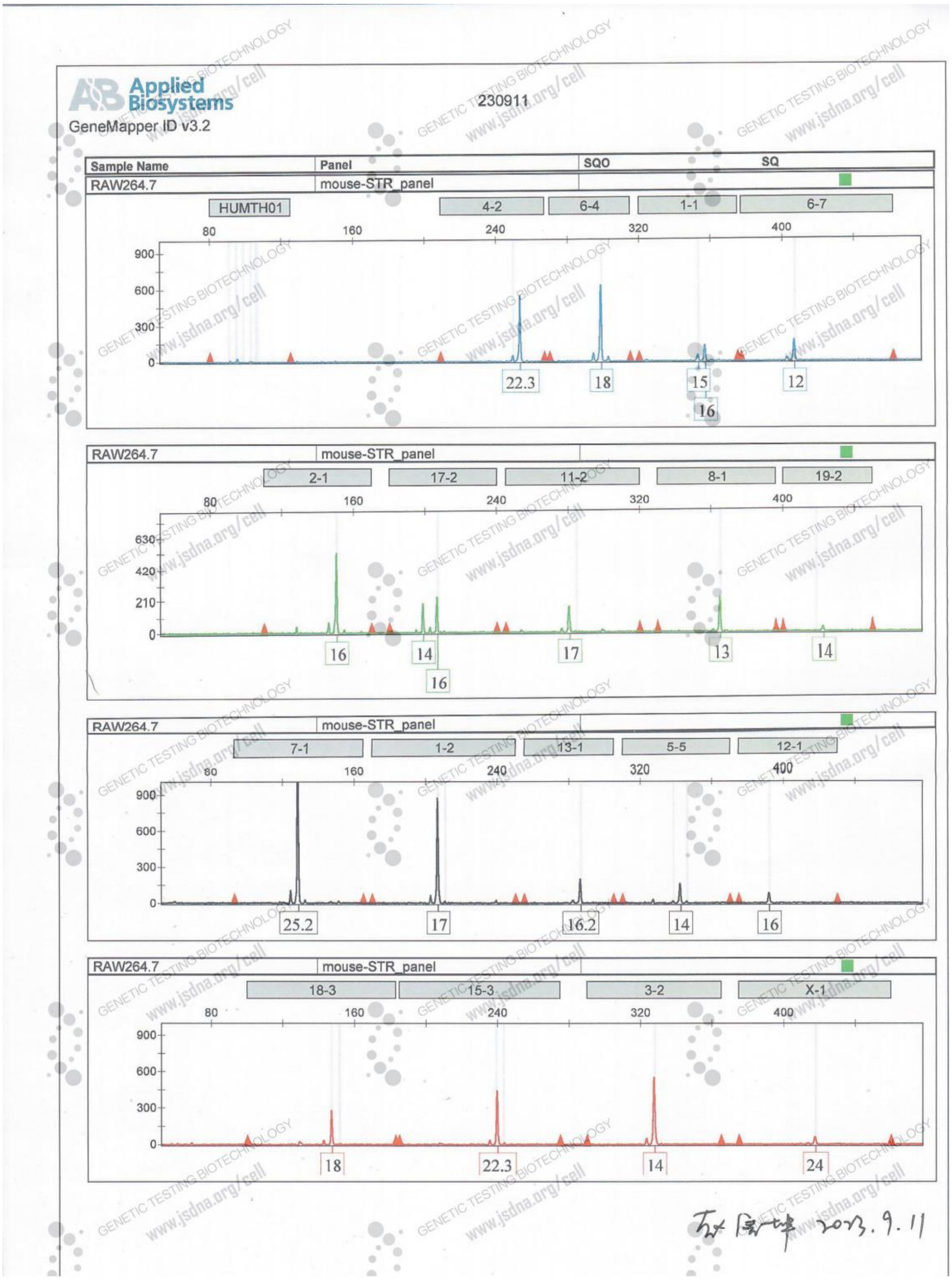

Figure S3. Results of authenticity verification of RAW 264.7 cell lines.

Eighteen short tandem repeat (STR) loci were amplified using multiplex PCR. One additional marker (Human TH01) was used to screen for the presence of human species. The cell line sample was processed using the ABI Prism® 3130 XL Genetic Analyzer. Data were analyzed using Gene Mapper® ID v3.2 software (Applied Biosystems). Appropriate positive and negative controls were run and confirmed for each sample submitted. Cell lines were authenticated using Short Tandem Repeat

(STR) analysis as described in Almeida JL et al., Interlaboratory study to validate a STR profiling method for intraspecies identification of mouse cell lines [1].
